# The synthesis of two-dimensional MoS_2_ nanosheets with enhanced tribological properties as oil additives†

**DOI:** 10.1039/c7ra12897e

**Published:** 2018-03-06

**Authors:** Meirong Yi, Chenhui Zhang

**Affiliations:** State Key Laboratory of Tribology, Tsinghua University Beijing 100084 China chzhang@tsinghua.edu.cn

## Abstract

The use of MoS_2_ nanosheets as oil additives has been proved effective to reduce friction and wear. Furthermore, it has been suggested that the synthesis of MoS_2_ nanosheets with an ultrathin structure could benefit the friction and wear reduction, as they would penetrate into the contact area easily. In this paper, two-dimensional MoS_2_ nanosheets were successfully fabricated by a solvothermal method with the aid of oleylamine. Meanwhile, the synthesized MoS_2_ nanosheets exhibited perfect dispersing stability in paraffin oil, due to the surface modification by oleylamine molecules. The friction and wear properties of the synthesized MoS_2_ nanosheets as oil additives were investigated using a ball-on-disk tribotester. The results showed that the two-dimensional MoS_2_ nanosheets exhibited enhanced friction-reducing and antiwear behaviors as compared to the multilayered MoS_2_ nanosheets. The prominent tribological performance of the two-dimensional MoS_2_ nanosheets was attributed to the formation of a thick tribofilm inside the wear tracks, which was confirmed by XPS analyses of the rubbing interfaces.

## Abstract 2

Two-dimensional MoS_2_ nansheets (2D MoS_2_) with enhanced tribological properties were successfully fabricated with the aid of oleylamine.

## Graphical abstract

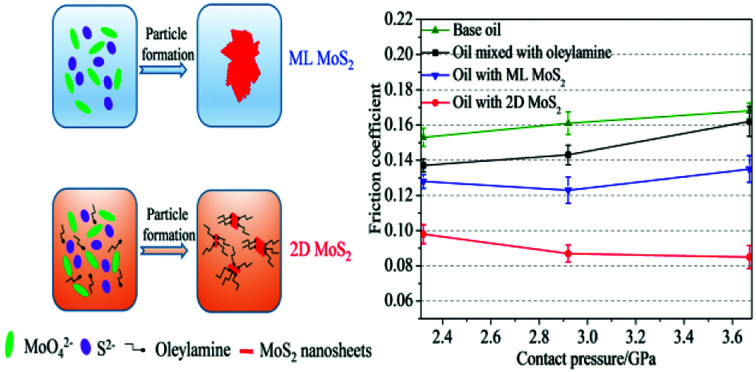

## 1. Introduction

The discovery and identification of graphene in 2004 (ref. 1) has evoked an explosion of interest in other two-dimensional (2D) nanomaterials.^2^ As a typical example, 2D molybdenum disulfide (MoS_2_) has a structure analogous to graphene, in which two close-packed sulfur atom layers sandwich one molybdenum atom layer and these layers are held together by van der Waals forces.^3^ Due to their extremely thin structure, the 2D MoS_2_ nanosheets exhibit unique mechanical, electrical, thermal and optical properties,^4^ making them significantly different from their bulk counterparts and also resulting in their application in catalysis,^5,6^ electronics,^7^ photonics,^8^ energy storage,^9,10^ and sensors.^11^

Over the past few decades, MoS_2_ nanoparticles with various morphologies, such as nanosheets,^12^ flower-like structure,^13^ sphere-like structure^14^ and fullerene-like structure,^15^ have been used as oil additives. Meanwhile, the lubrication mechanisms of MoS_2_ nanoparticle in oils have been extensively studied and suggested as rolling, interlayer sliding and exfoliation–material transferring.^16^ In particular, the formation of a tribofilm composed of MoS_2_ molecules has been emphasized as the key mechanism for decreasing the friction and wear.^17^ In addition, Chen *et al.*^18^ recently reported the excellent extreme pressure property of ultrathin MoS_2_ nanosheets as oil additives. They suggested that the ultrathin shape of MoS_2_ nanosheets could ensure the entrance of the nanosheets into the rubbing interfaces. Therefore, it is desirable to fabricate 2D MoS_2_ nanosheets to be used as oil additives, as they could be penetrated into the contact area easily.

According to the relevant literatures, it is found that the existing research on 2D MoS_2_ nanosheets has largely relied on the exfoliation method,^19^ chemical vapor deposition method^20^ or solvothermal route.^21^ For example, Dong *et al.*^22^ fabricated 2D MoS_2_ nanosheets *via* the sonication assisted liquid exfoliation in organic solvents. However, the prepared ultrathin MoS_2_ nanosheets had a large distribution in particle size. Meanwhile, Bang *et al*.^23^ proposed that the exfoliation method may induce a phase change of the bulk MoS_2_. In addition, there are other disadvantages associated with the exfoliation method, such as, the time-consuming process and the aggregation of the exfoliated nanosheets after the solvent being removed.^24,25^ As to the chemical vapor deposition method, it needs rigorous experimental conditions, such as high temperature, high vacuum and special substrates, which could limit the practical application of 2D MoS_2_ nanosheets.^26^ By comparison, the solvothermal method could allow the production of large quantities of 2D MoS_2_ nanosheets along with the advantages of low cost, simple operation, and minimal environmental impact.^27,28^

Therefore, the solvothermal method was employed in this work to synthesize 2D MoS_2_ nanosheets by using heptamolybdate tetrahydrate and thiourea as precursors in oleylamine. The tribological behavior of the 2D MoS_2_ nanosheets as oil additives was investigated by a ball-on-disk tribotester. This objective of this paper is to explore the potential of 2D MoS_2_ nanosheets to serve as oil additives.

## 2. Experimental section

### 2.1 Materials

Ammonium heptamolybdate tetrahydrate ((NH_4_)_6_Mo_7_O_24_·4H_2_O), thiourea ((NH_2_)_2_CS) were purchased from Sinopharm Chemical Reagent Co., Ltd. (China). Oleylamine (C_18_H_37_N) was purchased from Sigma Aldrich. All chemicals were used as received without further treatment.

### 2.2 Materials preparation and characterization

Two-dimensional MoS_2_ nanosheets (2D MoS_2_) were synthesized through a solvothermal route. In a typical synthesis, 0.618 g heptamolybdate tetrahydrate ((NH_4_)_6_Mo_7_O_24_·4H_2_O) and 1.14 g thiourea ((NH_2_)_2_CS) were added to 30 mL of oleylamine solution. The mixture was then heated to 130 °C under a N_2_ flow and held for 30 min, enabling the (NH_4_)_6_Mo_7_O_24_·4H_2_O and N_2_H_4_CS to be dissolved completely. After that, the solution was transferred into a 50 mL autoclave and kept at 200 °C for 12 h. The black precipitates were collected by centrifugation, washed with hexane and ethanol, and dried in vacuum at 60 °C for 6 h. For comparison purpose, multilayered MoS_2_ nanosheets (ML MoS_2_) were obtained *via* the same procedure, while using pure distilled water, instead of oleylamine, as the reaction solvent. The transmission electron microscopy (TEM, JEM-2010) was used to observe the morphology of the synthesized samples. Power X-ray diffraction (XRD, Thermo Fisher ESCALAB 250Xi Diffractometer with Cu Kα radiation) was employed to identify their phase structures. Raman spectra were collected on a Horiba Jobin Yvon Raman spectrometer with a laser excitation wavelength of 514.5 nm. Fourier transform infrared (FTIR) spectra were obtained on a TGA 2050 infrared spectrometer at a spectral resolution of 4 cm^−1^. Thermogravimetric analysis (TGA, SDT Q600) was conducted under air flow with a heating rate of 10 °C min^−1^. X-ray photoelectron spectroscopy (XPS) was carried out on a PHI Quantera X-ray photoelectron spectrometer.

### 2.3 Friction tests

The synthesized MoS_2_ samples were distributed into the base oil (liquid paraffin) by 30 min ultrasonication. A ball-on-disk tribotester (Optimal SRV 4) was used to examine the tribological behavior of these oils. The friction tests were conducted at stroke of 2 mm and frequency of 50 Hz. Each friction test lasted for 1 hour and was repeated three times. A commercially available AISI-52100 steel ball with diameter of 10 mm, hardness of 61–63 HRC and surface roughness of 25 nm was chosen as the upper specimen. The counterpart was an AISI-52100 steel disk with diameter of 24 mm, height of 7.88 mm and surface roughness of 50 nm. After friction tests, the topographies of the wear tracks on the disks were examined by SEM. Meanwhile, the depth of the wear tracks on the disks was measured using a white-light interference profilometer (Phase Shift MicroXAM-3D). The XPS technique was also used to investigate the tribo-reaction film inside the wear tracks.

## 3. Results and discussion

### 3.1 Characterizations of the synthesized samples

**Fig. 1:**
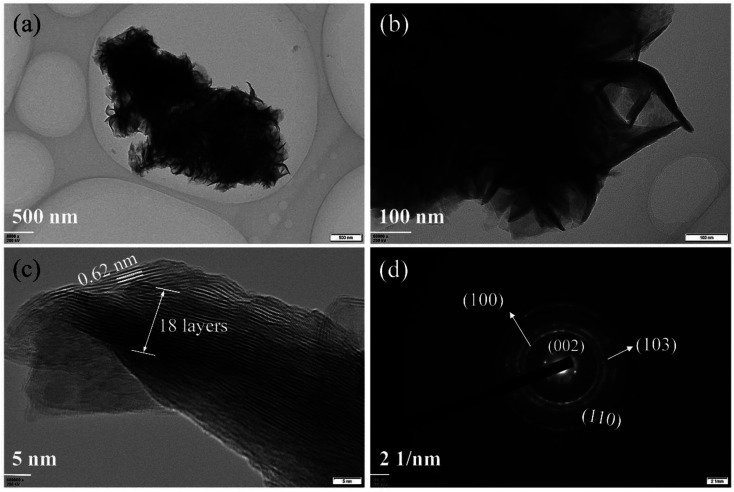
(a and b)TEM images, (c) HRTEM image and (d) SAED pattern of the synthesized ML MoS_2_.

**Fig. 2:**
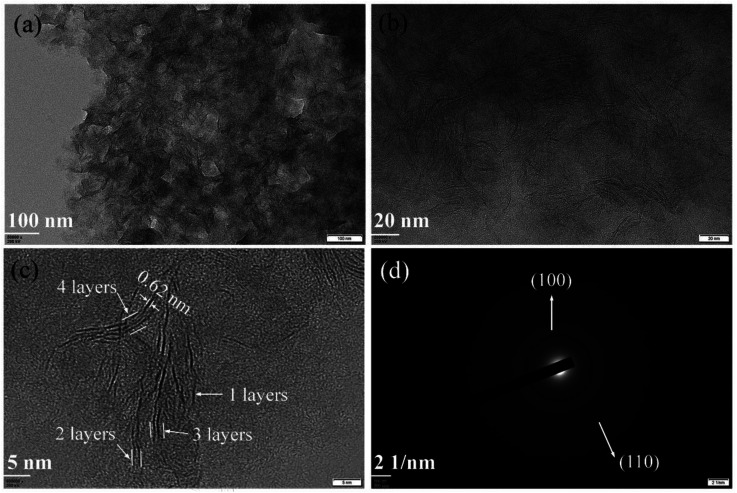
(a and b)TEM images, (c) HRTEM image and (d) SAED pattern of the synthesized 2D MoS_2_.

The morphologies of synthesized samples were observed by TEM. As Fig. 1a and b shown, the multilayered MoS_2_ nanosheets (ML MoS_2_) present a cluster-like shape and consist of accumulated nanosheets. It is also found that the nanosheets in the ML MoS_2_ are stacked densely with an interlayer separation of 0.62 nm (Fig. 1c). The SAED pattern of the ML MoS_2_ (Fig. 1d) consists of four diffraction rings that could be indexed to the (002), (100), (103) and (110) planes of MoS_2_.^29^ The morphology of the two-dimensional MoS_2_ nanosheets (2D MoS_2_) was also examined by TEM (Fig. 2). As Fig. 2a and b shown, the obtained 2D MoS_2_ is comprised of ultrathin nanosheets with lateral size ranging from 20 nm to 30 nm. It is also found that a fraction of the nanosheets are slightly curved, similar to the phenomenon reported by Savjani *et al.*^30^ The reason is that such ultrathin nanosheets are unstable and easy to form closed structures by rolling up to eliminate dangling bonds at the edges.^31^ The extremely thin structure of the 2D MoS_2_ is clearly shown in Fig. 2c, as it observes that each nanosheet is composed of one to four layers. The interlayer distance measured from Fig. 2c is 0.62 nm, consistent with the (002) *d*-spacing for the hexagonal MoS_2_. The SAED pattern illustrates the polycrystalline nature of the 2D MoS_2_, as no clear spots corresponding to the (100) and (110) planes of MoS_2_ observed, only highly diffused bands (Fig. 2d).

**Fig. 3:**
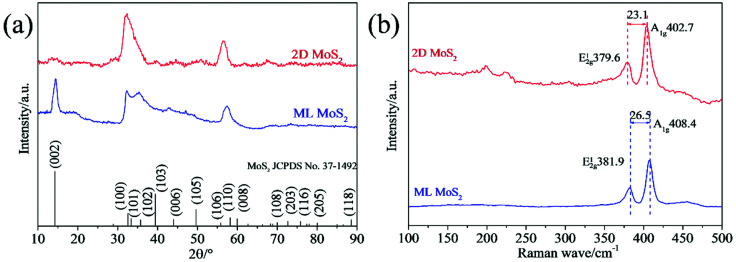
(a) XRD patterns and (b) Raman spectra of the synthesized MoS_2_ samples.

The phase structures of the synthesized samples were investigated by the XRD. As Fig. 3a shown, all the diffraction peaks of the two samples can be indexed to the hexagonal MoS_2_ (JCPDS no. 37-1492). Meanwhile, the ML MoS_2_ displays a strong and sharp (002) diffraction peak, suggesting the well-layered structure of this sample. However, there is no (002) diffraction peak detected in the 2D MoS_2_. It indicates that the stacking of the (002) plane in the 2D MoS_2_ is significantly inhibited and extremely thin MoS_2_ nanosheets were obtained. In addition, according to the Bragg's equation, the *d*-spacing for the (002) plane in the ML MoS_2_ is calculated to be 0.62 nm, consistent with the TEM analysis (Fig. 1c). Fig. 3b showed the Raman spectra of the two samples. The Raman test of MoS_2_ should presents two kinds of optical phonon modes (E^1^_2g_ and A_1g_), where E^1^_2g_ is the in-plane sulfur–molybdenum vibrations and A_1g_ corresponds to the out-plane sulfur vibrations. As Fig. 3b shown, the E^1^_2g_ and A_1g_ modes of the ML MoS_2_ are detected at 381.9 and 402.7 cm^−1^, respectively. For the 2D MoS_2_, the frequencies of the E^1^_2g_ and A_1g_ modes are found at the positions of 379.6 cm^−1^ and 408.4 cm^−1^, respectively. It has been reported before that the space between the Raman modes of E^1^_2g_ and A_1g_ could decrease as the layer number of MoS_2_ nanosheets decreases.^32^ From Fig. 3b, it is observed that the ML MoS_2_ exhibits a separation of 26.5 cm^−1^ between the Raman modes of E^1^_2g_ and A_1g_. While the 2D MoS_2_ shows the two Raman modes separated by 23.1 cm^−1^. This indicates that the numbers of layers in the 2D MoS_2_ have a reduction as compared with the ML MoS_2_. In addition, the value of 23.1 cm^−1^ conforms to the peak separation observed for tri-layered MoS_2_ nanosheets.^32^

**Fig. 4:**
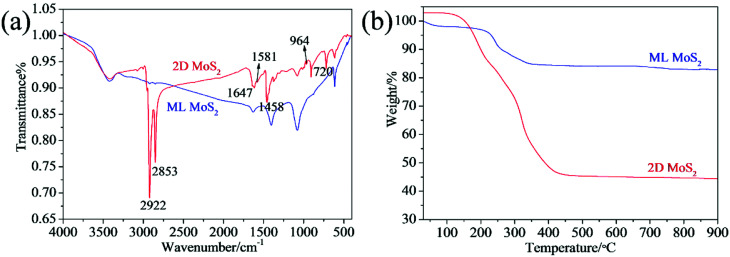
(a) FTIR spectra; (b) TGA results of the synthesized MoS_2_ samples.

Fig. 4a presents the FTIR spectra of the two MoS_2_ samples. The absorptions at 1402, 1080, 875 and 607 cm^−1^ can be attributed to the MoS_2_.^33^ Comparing with the ML MoS_2_, several new bands are observed in the FTIR spectrum for the 2D MoS_2_. The bands at 2922 cm^−1^ and 2853 cm^−1^ can be assigned, respectively, to the asymmetric and symmetric vibrations of the C–H in oleylamine. The band at around 1647 cm^−1^ can be attributed to the stretching vibration of the C=C present in oleylamine.^34^ The peaks at 1458 cm^−1^ and 722 cm^−1^ can be assigned to the bending vibrations of the C–H and C–C from oleylamine. In addition, there is characteristics signal of the amine group: the peak at 1581 cm^−1^ due to the –NH_2_ scissoring mode and the peak due to –NH_2_ bending mode at 964 cm^−1^.^28^ The FTIR results confirm that the surfaces of the 2D MoS_2_ were attached by oleylamine molecules. Thermogravimetric analysis (TGA) was further carried out to determine the amount of oleylamine present in the 2D MoS_2_ (Fig. 4b). For the ML MoS_2_, the main weight loss (11.2 wt%) takes place around the temperature range of 200 to 380 °C, due to the oxidation of MoS_2_ into MoO_3_ in air. There is also a weight loss (2 wt%) below 100 °C, which can be attributed to the removal of absorbed water. For the 2D MoS_2_, after oleylamine molecules attached onto their surfaces, the total weight loss increases to 56.2 wt%. From Fig. 4b, it can be estimated that the amount of oleylamine attached onto the surfaces of the 2D MoS_2_ is about 51.2 wt%.

**Fig. 5:**
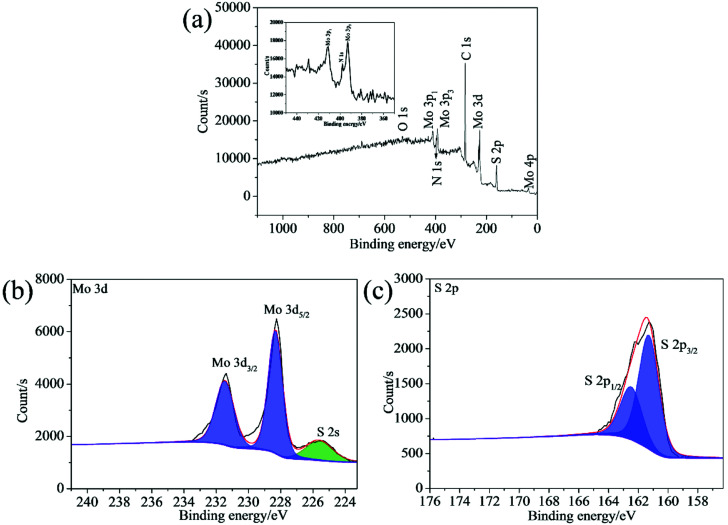
(a) XPS survey spectrum and high-resolution XPS spectra of (b) Mo 3d; (c) S 2p for the synthesized 2D MoS_2_.

The chemical composition of the synthesized samples was investigated using the XPS. The survey spectrum indicates the coexistence of C, Mo, S and N elements in the 2D MoS_2_ (Fig. 5a). The N element could come from oleylamine molecules attached onto the surfaces of the 2D MoS_2_. The high resolution of Mo 3d displays two peaks at 231.27 and 228.13 eV, which can be ascribed to the Mo 3d_3/2_ and Mo 3d_5/2_ doublet (Fig. 5b). The peak at the position of about 225.41 eV in the Mo 3d spectrum belongs to the S 2s. The S 2p spectrum can be deconvoluted into two peaks at 162.28 and 161.09 eV, corresponding to the S 2p_1/2_ and S 2p_3/2_ orbital (Fig. 5c). The binding energies of the Mo 3d and S 2p are consistent with the reported values for the MoS_2_ particles prepared by other researchers,^35,36^ confirming the expected chemical states of Mo^4+^ and S^2−^ in the 2D MoS_2_. The binding energies of the Mo 3d and S 2p in the ML MoS_2_ also correspond to the expected values for MoS_2_ particles (Fig. S1†).

**Fig. 6:**
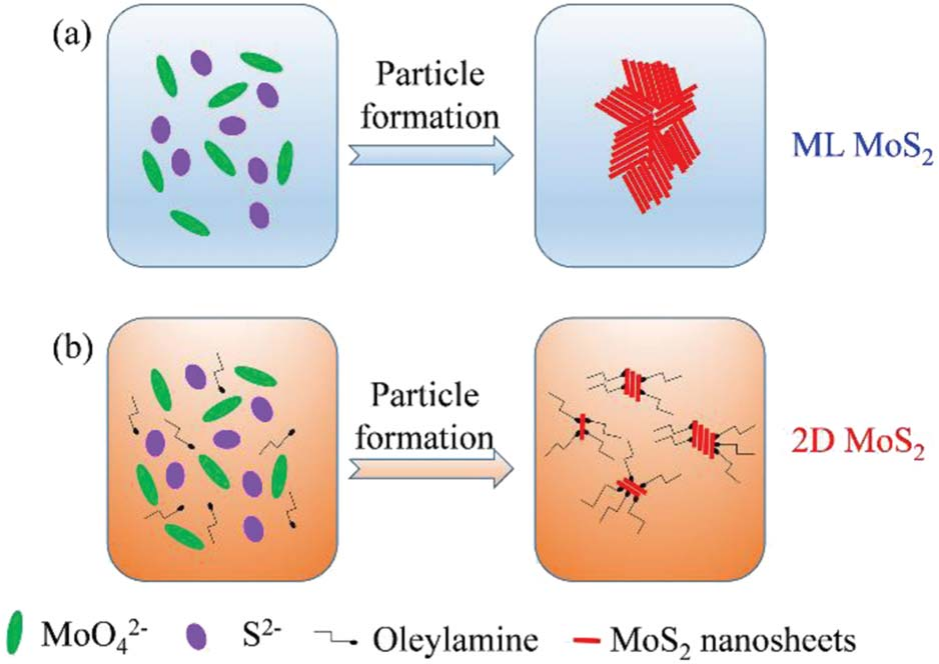
The reasonable formation procedure for the 2D MoS_2_.

Based on the above results, the reasonable formation procedure for the 2D MoS_2_ is shown in Fig. 6. When using the pure distilled water as the reaction solvent, the MoS_2_ nanosheets could aggregate together to form cluster-like structures. In this case, multilayered MoS_2_ nanosheets (ML MoS_2_) were obtained. While in the case of employing oleylamine as the reaction solvent, the growth of MoS_2_ grains were wrapped around by oleylamine molecules. Oleylamine is known as a ligand that binds tightly to the metal nanoparticles surfaces.^34^ Therefore, oleylamine molecules were attached onto the surfaces of MoS_2_ nanosheets to inhibit the growth of the nanosheets in the synthetic route. In addition, the long carbon chain of oleylamine can provide great steric hindrance to prevent the aggregation of the MoS_2_ nanosheets. As a result, oleylamine-modified MoS_2_ nanosheets with single or few layers were obtained.

### 3.2 Tribological property

**Fig. 7:**
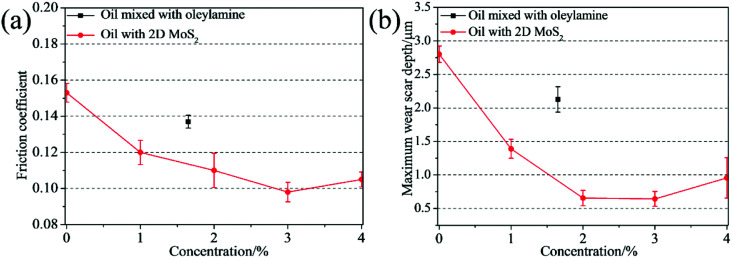
(a) Friction coefficients and (b) maximum wear scar depth as functions of the 2D MoS_2_ concentration in oils.

In order to confirm the merits of the 2D MoS_2_ as oil additives, the tribological behaviors of the oils dispersed with 2D MoS_2_ or ML MoS_2_ were investigated. Fig. 7a shows the friction coefficient of the oils with different 2D MoS_2_ concentrations at the load of 100 N (corresponding to the initial maximum Hertz contact pressure of 2.32 GPa). It is observed that there is an obvious reduction in the friction coefficient when the oil with 2D MoS_2_ was used for lubrication. Meanwhile, it is found that the friction coefficient first decreases quickly and then rises slightly as the concentration of the 2D MoS_2_ increasing. The lowest friction coefficient (0.098) is obtained at the 2D MoS_2_ concentration of 3 wt%. In addition, the amount of oleylamine in the oil sample with 3 wt% 2D MoS_2_ is about 1.5 wt% through the TGA result (Fig. 4b). Therefore, the tribological behavior of the oil mixed with 1.5 wt% oleylamine was also tested. In this case, a friction coefficient of 0.137 is obtained and this value has no obvious variation from that of the base oil (0.153). This finding confirms that the excellent friction-reduction effect of the 2D MoS_2_ comes from the MoS_2_ nanosheets, instead of oleylamine. The antiwear property of these oil samples was analyzed using the maximum wear scar depth on the lower disks (Fig. 7b). The result is similar to the relationship between the friction coefficient and the 2D MoS_2_ concentration. The minimal wear can be found at the 2D MoS_2_ concentration of 3 wt%, and beyond this concentration the wear slightly rise. Fig. S2† shows the friction coefficient and maximum wear scar depth measured under the lubrication of the oil with ML MoS_2_. The friction coefficient and wear could decrease to some extent after adding the ML MoS_2_ into the base oil. However, the extent of decline in the friction coefficient and wear is far less than that lubricated with the 2D MoS_2_. It has been known that one of the key prerequisites for using nanoparticles as oil additives is their dispersing stability in oils. Fig. S3† shows the photos of the oil dispersed with 1.5 wt% ML MoS_2_ or 3 wt% 2D MoS_2_ after standing 7 days. No obvious precipitate is observed in the photo of the oil with 2D MoS_2_ (Fig. S3b†). This can be attributed to the surface modification of the 2D MoS_2_ by oleylamine molecules (Fig. 4). The result indicates that 2D MoS_2_ can be used as oil additives without the obstacle of poor dispersing stability in oils.

**Fig. 8:**
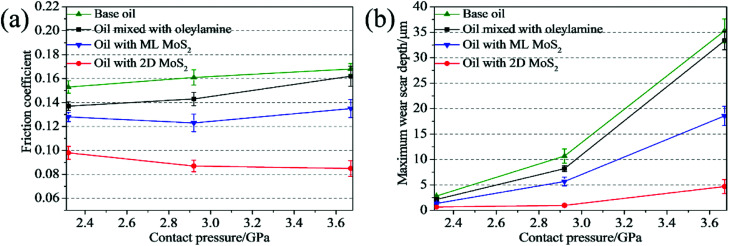
(a) Friction coefficients and (b) maximum wear scar depth as functions of the contact pressure.

The effect of the load on the tribological behaviors of the oils was also investigated under the different loads of 100, 200 and 400 N, corresponding to initial maximum Hertz contact pressures of 2.32, 2.92 and 3.67 GPa, respectively. The oils with 3 wt% 2D MoS_2_ or 1.5 wt% ML MoS_2_ were selected as testing samples. From Fig. 8a, it is observed that the oil with 2D MoS_2_ exhibits a relatively smaller friction coefficient than the others under all testing loads. In addition, the friction-reducing behavior of the oil with 2D MoS_2_ gradually strengthens with the increasing contact pressure. Specially, comparing with the base oil, the friction coefficient of the oil with 2D MoS_2_ decreases 35.95% at the relatively low contact pressure of 2.32 GPa. Whereas its friction coefficient becomes much lower than that of the base oil when the contact pressure increases to 2.92 GPa (45.96%) and 3.67 GPa (49.40%). Meanwhile, it is observed that there is a reduction in the wear scar depth after the addition of MoS_2_ nanosheets into the base oil at any given load (Fig. 8b). While the wear reduction for the oil with 2D MoS_2_ is more remarkable as compared to the oil with ML MoS_2_. Further, it is also observed that wear resistance ability of the oil with 2D MoS_2_ enhances with the increasing contact pressure. As Fig. 8b shown, the maximum wear scar depth for the oil with 2D MoS_2_ is reduced by 3 times with respect to the base oil at lower contact pressure of 2.32 GPa. However, this behavior is amplified at higher contact pressure (2.92 or 3.67 GPa), where the maximum wear scar depth is at least reduced by 6 times.

**Fig. 9:**
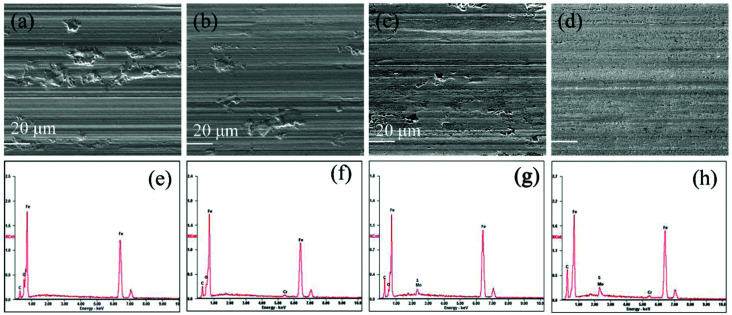
SEM and EDS analyses of the wear tracks lubricated by: (a and e) base oil; (b and f) oil mixed with oleylamine; (c and g) oil with ML MoS_2_; (d and h) oil with 2D MoS_2_.

After friction tests (at 2.92 GPa), the topographies of the wear tracks on the lower disks were analyzed by the SEM. As shown in Fig. 9a, the surface of the wear track lubricated by the base oil is evidently rough and dominated by severe abrasion. This attributes to the poor antiwear property of the pure paraffin oil. For the wear track lubricated with oleylamine in the base oil, the worn surface is as well very rough and displays many deep furrows (Fig. 9b). This suggests that oleylamine has no obvious function in improving the antiwear property of the base oil. Similarly, there are obvious scratches and deep grooves observed on the surface lubricated with the ML MoS_2_ (Fig. 9c). However, a relatively uniform and smooth worn surface was obtained when the oil with 2D MoS_2_ was used (Fig. 9d). In addition, only minor pits instead of scratched furrows are observed inside the wear track. These results indicate that the 2D MoS_2_ performs more excellent in improving the antiwear ability of the base oil as compared to the ML MoS_2_. Meanwhile, the peaks from Mo and S atoms in the EDS spectrum (Fig. 9g and h) indicate the formation of a MoS_2_-based tribofilm inside the wear track lubricated with MoS_2_ particles.

**Fig. 10:**
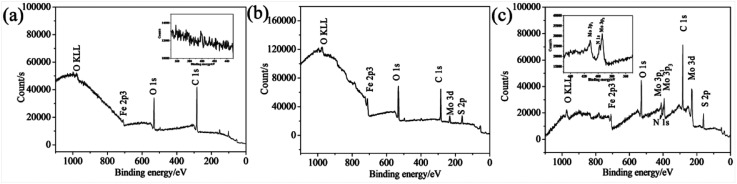
XPS survey spectrum for the wear tracks lubricated by: (a) oil mixed with oleylamine; (b) oil with ML-MoS_2_; (c) oil with 2D MoS_2_.

**Fig. 11:**
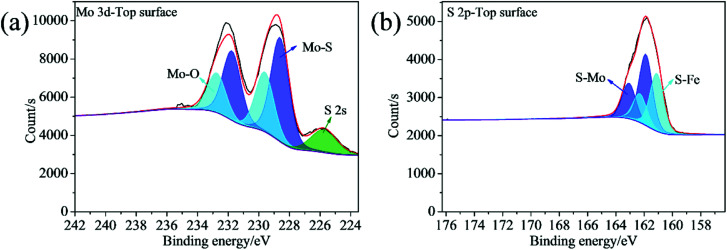
XPS spectra of (a) Mo 3d and (b) S 2p for the wear track lubricated with the 2D MoS_2_.

In order to confirm the formation of tribofilm inside the wear tracks and its composition, the XPS analyses were carried out. The elements of C, O, Fe are detected inside the wear track lubricated with oleylamine in the base oil (Fig. 10a). It can be seen that oleylamine cannot tightly adsorbed onto the rubbing surfaces, as no N element detected inside the wear track. For the wear track lubricated with the ML MoS_2_, the observed elements include C, O, Fe, and a trace amount of Mo and S (Fig. 10b). As to the wear track lubricated with the 2D MoS_2_, the elements including C, O, Fe, Mo, S as well as N are detected (Fig. 10c). The N element could from oleylamine molecules attached onto the surfaces of the 2D MoS_2_. The high-resolution spectra of Mo 3d and S 2p recorded from the wear track lubricated with the 2D MoS_2_ are shown in Fig. 11. The Mo 3d_5/2_ is composed of a main peak at 228.61 eV corresponding to the MoS_2_, a small peak at 229.60 eV corresponding to the Mo–O species, which suggests that the 2D MoS_2_ was transferred into contact surfaces and part of them was oxidized. As to the S 2p_3/2_ peak, a peak at 161.88 eV can be assigned to MoS_2_ and another peak at 161.11 eV can be attributed to the S–Fe species. The presence of the S–Fe species illustrates that the MoS_2_ adhered onto the rubbing surfaces through the Fe–S covalent bond. Meanwhile, the O 1s and Fe 2p3 peaks (Fig. S4†) suggests the presence of the oxide species (O–Fe and O–Mo) inside the tribofilm. The Mo 3d and S 2p XPS spectra recorded from the wear track lubricated with the ML MoS_2_ are shown in Fig. S5,† which are similar to those lubricated with the 2D MoS_2_.

**Fig. 12:**
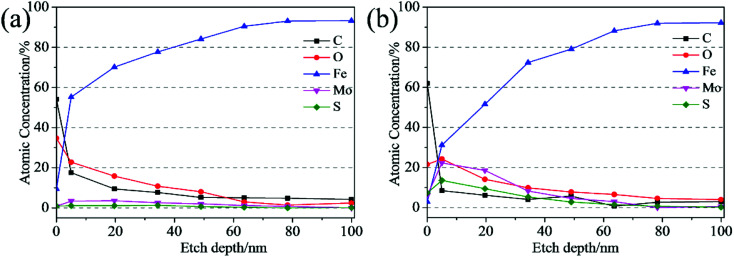
XPS depth profile of the wear track lubricated by: (a) oil with ML MoS_2_; (b) oil with 2D MoS_2_.

The XPS depth-profiling technique using argon ion sputtering was employed to analyze the thickness of the tribofilm. Fig. 12 shows the depth profiles of all elements detected inside the tribofilm. As Fig. 12 shown, the iron signal is very weak at the top surface of the tribofilm, and then increases with the etch depth increasing. Finally, the iron content remains stable, which means the tribofilm was sputtered out completely and the steel substrate was reached. For the wear track lubricated with the ML MoS_2_ (Fig. 12a), the concentrations of Mo and S elements on the top surface are quite low (0.8 at% and 0.9 at%, respectively), which implies that the MoS_2_ tribofilm did not completely covered the wear track. Meanwhile, it is observed that the concentrations of Mo and S elements inside the tribofilm formed from the oil with ML MoS_2_ are very low throughout the etch depth and decrease fast during the initial 19 nm. As to the tribofilm formed from the oil with 2D MoS_2_ (Fig. 12b), the Mo and S elements on the top surface of the wear track are in relatively high concentration (6.0 at% and 7.3 at%, respectively). Moreover, it is observed that there is a significant increase in the concentrations of Mo and S elements (22.3 at%, 13.6 at%, respectively) after 5 nm depth of etching. From Fig. 12b, it can be estimated that the thickness of the tribofilm formed from the oil with 2D MoS_2_ is about 78 nm. Meanwhile, the Mo element inside the tribofilm is found to exist in two forms: the MoS_2_ and the Mo–O species (Fig. S6a†). The S element inside the tribofilm is presented by the two chemical states of the MoS_2_ and the S–Fe species (Fig. S6b†). It can be seen that the tribofilm formed from the oil with 2D MoS_2_ appears much thicker with higher concentrations of Mo and S, comparable to that lubricated with the ML MoS_2_.

## 4. Discussion

The minimum oil film thickness can be predicted *via* the Hamrock–Dowson equation:^37^1
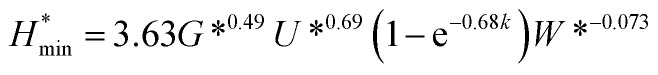
2
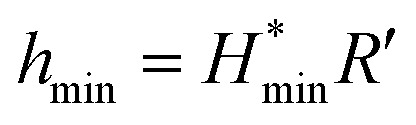

3 G* = *αE*′, U* = *η*_0_*U*/(*E*′*R*′), W* = *W*/(*E′R′*^2^)

where *h*_min_ is the minimum oil film thickness, *α* is the pressure–viscosity of the lubricant (2.0 × 10^−8^ m^2^ N^−1^), *E*′ is the effective modulus of elasticity (208 GPa), *U* is the entrainment velocity (0.1 m s^−1^), *η*_0_ is the ambient viscosity of the lubricant (0.024 Pa s), *R*′ is the effective radius, *k* = 1.03.

It can be seen from Table 1, at the beginning of the test, the minimum oil film thickness in the contact area is 14.64, 13.92 and 13.23 nm, respectively, for the tested loads of 100, 200 and 400 N. The surface roughness of the ball and disk is 25 nm and 50 nm, respectively. It can be deduced that the sliding surfaces are running in the boundary lubrication region. With the process of the tests, wear occurred both on the ball and the disk, could result an increase in the contact area as compared to the initial contact area, which accordingly lead to the reduction of the contact pressure. According to the diameter of the wear scar on the ball, the average contact pressure and the corresponding minimum oil film thickness in the contact region have been calculated (see Table 1). It can be seen that the oil film thickness is less than or close to 50 nm under all tested loads. Meanwhile, the surface roughness of the disk increased to larger than 300 nm after tests, which is much larger than the calculated film thickness. Therefore, it can be deduced that the friction pairs are still in the boundary lubrication region at the end of tests. The small distance between the friction pair could restrict the entry of the MoS_2_ nanoparticles into the contact area in boundary lubrication region. In this case, the ability of MoS_2_ nanoparticles to enter and stay in the contact area is the key factor to improve the tribological property of the oils. From Fig. 8, it is observed that the oil with 2D MoS_2_ presented better lubrication performance than the oils with ML MoS_2_. This could be attributed the extremely thin structure of the 2D MoS_2_, which benefits their transfer into the contact area in the boundary lubrication region and promotes the formation of a MoS_2_-based tribofilm inside the wear track.

**Table 1:** The calculated oil film thickness under different lubricating conditions

Lubricating conditions	Oil with ML MoS_2_			Oil with 2D MoS_2_		
Load/N	100	200	400	100	200	400
Initial maximum Hertz contact/GPa	2.32	2.92	3.67	2.32	2.92	3.67
Oil film thickness (beginning)/nm	14.64	13.92	13.23	14.64	13.92	13.23
Wear scar diameter of upper balls/μm	428	602	1026	388	501	603
Average contact pressure/MPa	695	703	484	846	1015	1401
Oil film thickness/nm	34.04	37.75	54.74	29.68	29.20	26.04

## 5. Conclusions

In summary, two-dimensional MoS_2_ nanosheets (2D MoS_2_) were obtained *via* a solvothermal method by using heptamolybdate tetrahydrate and thiourea as precursors in oleylamine. Because of its coating characteristics, oleylamine led to the formation of 2D MoS_2_ with single or few layers. Moreover, the surfaces of the 2D MoS_2_ were attached by oleylamine molecules, which ensured their perfect dispersing stability in oils. The friction reduction and wear resistance properties of the paraffin oil could be improved with the addition of multilayered or two-dimensional MoS_2_ nanosheets. However, the 2D MoS_2_ exhibited enhanced tribological property as compared to the multilayered MoS_2_ nanosheets, resulting in the reduction of friction coefficient and wear by 49.4% and 91.0%, respectively. The significantly better tribological property of the oil with 2D MoS_2_ was attributed to the formation of a thick MoS_2_-based tribofilm on the lubricated metal surfaces, as the extremely thin structure of the 2D MoS_2_ greatly benefited their transfer into the contact area. These results significantly contributed to the commercial utilization of 2D MoS_2_ as oil additives.

## Conflicts of interest

We declare that we do not have any commercial or associative interest that represents a conflict of interest in connection with the work submitted.

## Supplementary Material

RA-008-C7RA12897E-s001
