# Supplementary material for: The synthesis of two-dimensional MoS2 nanosheets with enhanced tribological properties as oil additives
Source: RSC Adv. 2018 Mar 6;8(17):9564–73. doi: 10.1039/c7ra12897e (PMC9078693; doi:10.1039/c7ra12897e)
Supplement: RA-008-C7RA12897E-s001 [file RA-008-C7RA12897E-s001.pdf]

Electronic Supplementary Material (ESI) for RSC Advances.
This journal is © The Royal Society of Chemistry 2018

Electronic Supplementary Information

The synthesis of two-dimensional MoS_2 nanosheets for enhanced tribological property as oil additives

Meirong Yi, Chenhui Zhang*

State Key Laboratory of Tribology, Tsinghua University, Beijing 100084, China

*Corresponding author: Chenhui Zhang

Email: chzhang@tsinghua.edu.cn

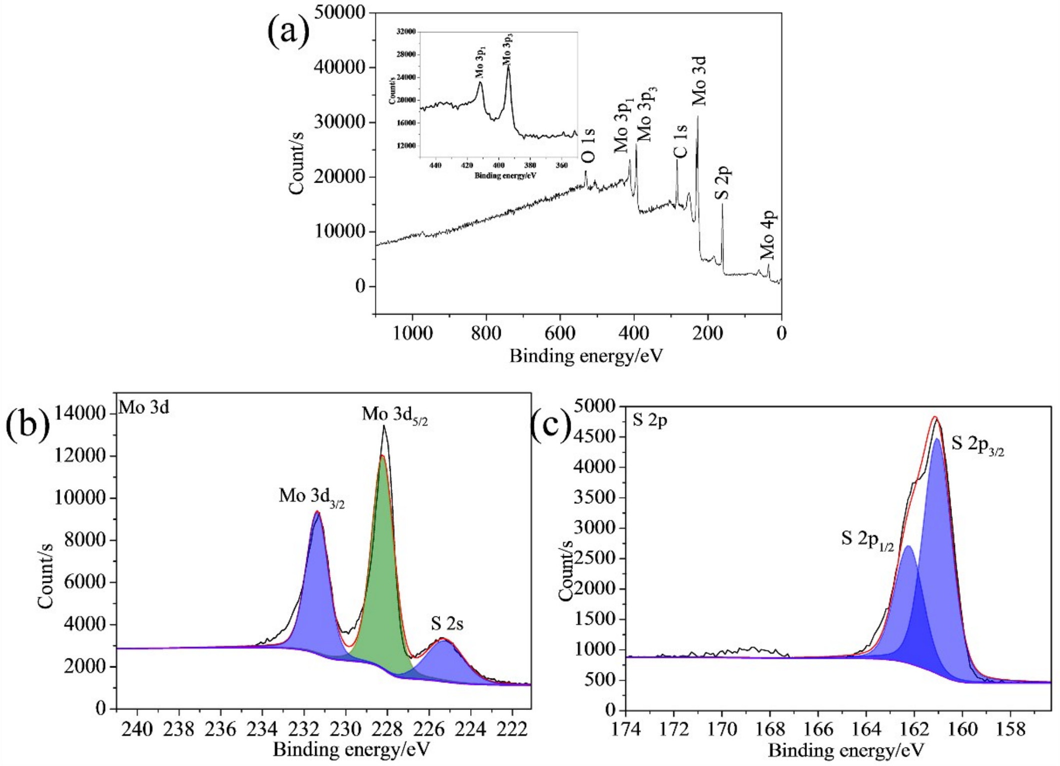

Fig. S1 (a) XPS survey spectrum and high-resolution XPS spectra of (b) Mo 3d; (c) S 2p for the synthesized ML MoS_2

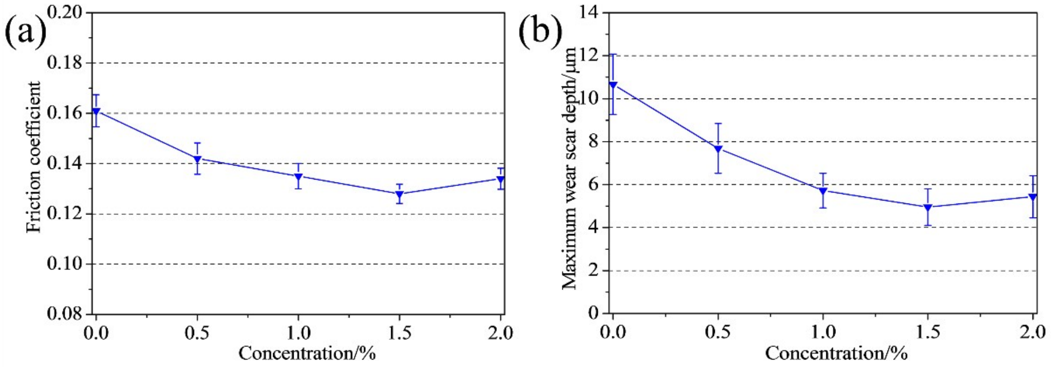

Fig. S2 (a) Friction coefficients and (b) maximum wear scar depth as functions of the ML MoS_2 concentration in oils

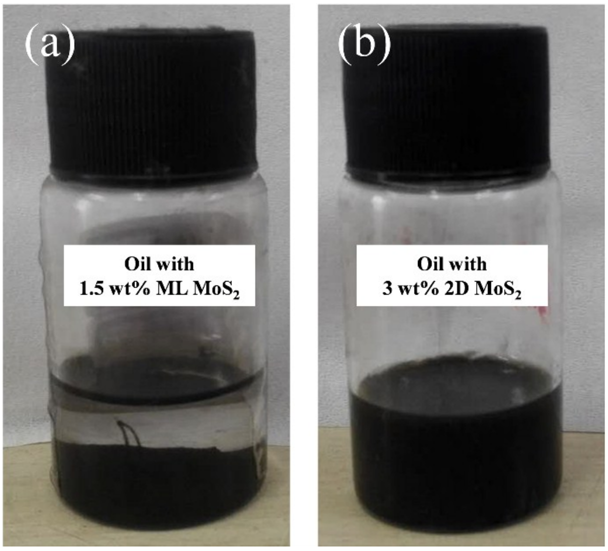

Fig. S3 Photos of the oils after standing 7 days

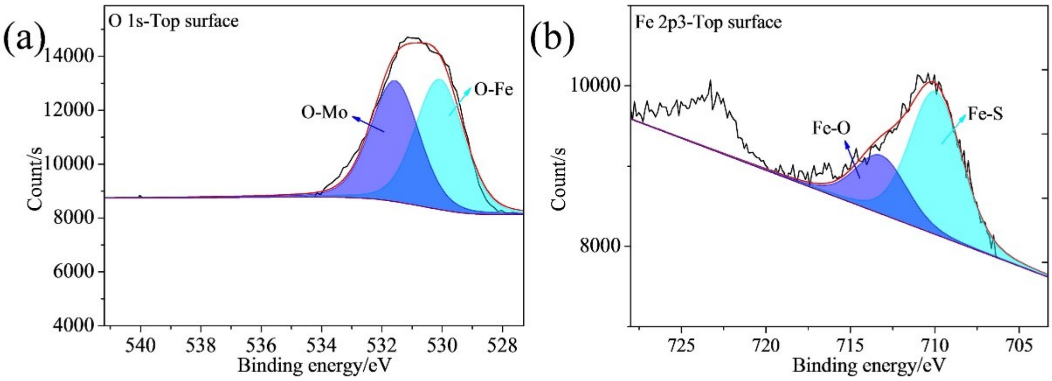

Fig. S4 XPS spectra of (a) O 1s and (b) Fe 2p3

for the wear track lubricated with the 2D MoS_2

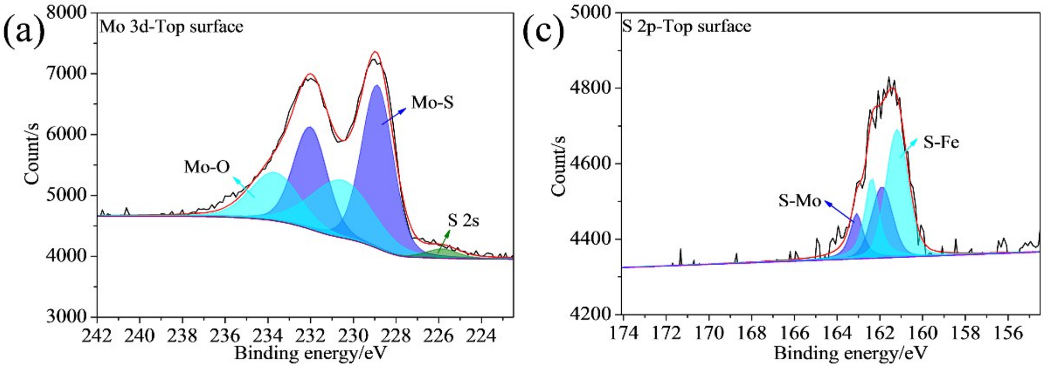

Fig. S5 XPS spectra of (a) Mo 3d and (b) S 2p for the wear track lubricated with the ML MoS_2

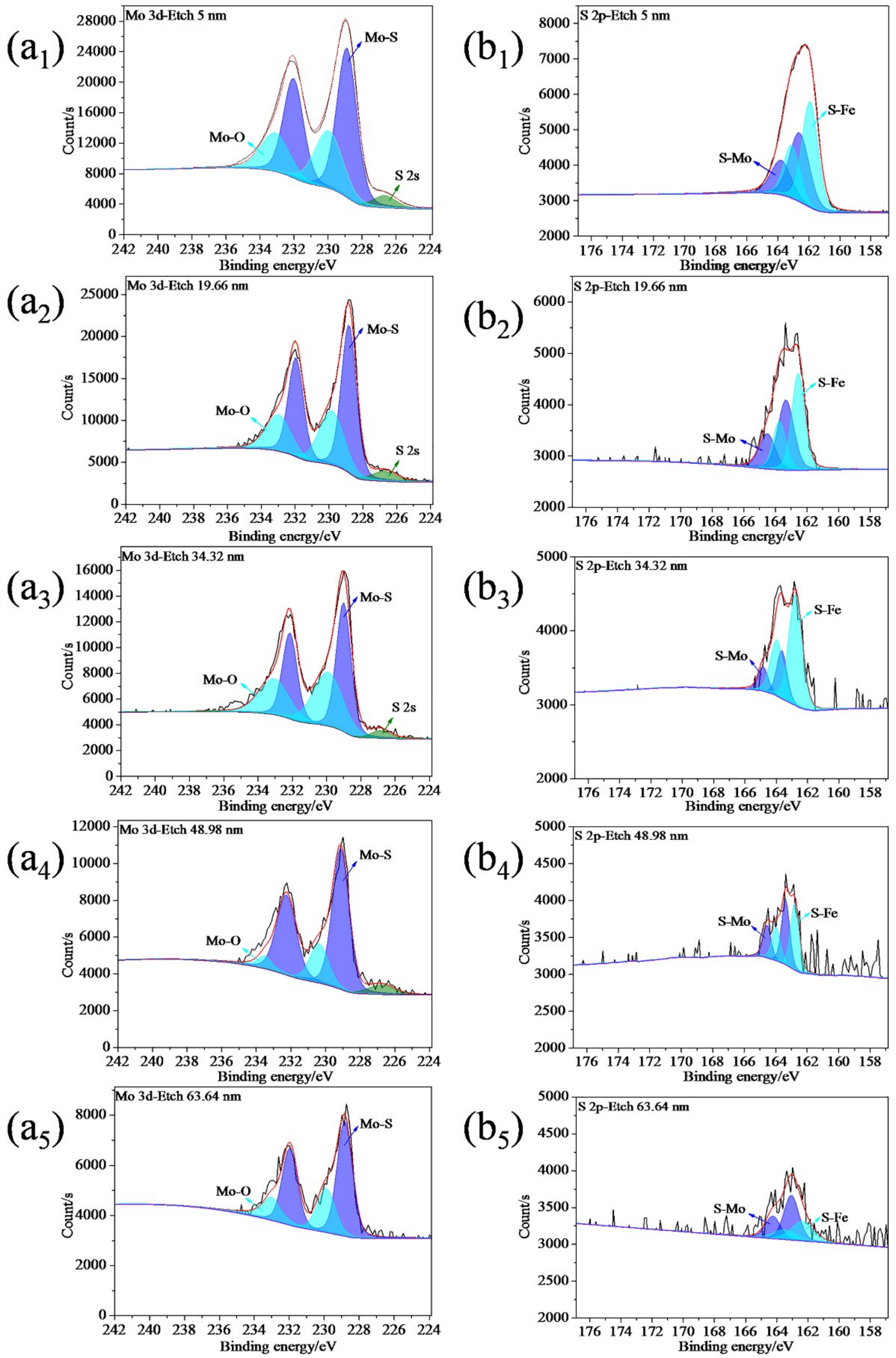

Fig. S6 XPS spectra of (a) Mo 3d and (b) S 2p with etching depth for the wear track lubricated with the 2D MoS_2
